# Dual Attention and Patient Similarity Network for drug recommendation

**DOI:** 10.1093/bioinformatics/btad003

**Published:** 2023-01-06

**Authors:** Jialun Wu, Yuxin Dong, Zeyu Gao, Tieliang Gong, Chen Li

**Affiliations:** School of Computer Science and Technology, Xi’an Jiaotong University, Xi’an 710049, China; Shaanxi Provincial Key Laboratory of Big Data Knowledge Engineering, Xi’an Jiaotong University, Xi’an 710049, China; School of Computer Science and Technology, Xi’an Jiaotong University, Xi’an 710049, China; Shaanxi Provincial Key Laboratory of Big Data Knowledge Engineering, Xi’an Jiaotong University, Xi’an 710049, China; School of Computer Science and Technology, Xi’an Jiaotong University, Xi’an 710049, China; Shaanxi Provincial Key Laboratory of Big Data Knowledge Engineering, Xi’an Jiaotong University, Xi’an 710049, China; School of Computer Science and Technology, Xi’an Jiaotong University, Xi’an 710049, China; Shaanxi Provincial Key Laboratory of Big Data Knowledge Engineering, Xi’an Jiaotong University, Xi’an 710049, China; School of Computer Science and Technology, Xi’an Jiaotong University, Xi’an 710049, China; Shaanxi Provincial Key Laboratory of Big Data Knowledge Engineering, Xi’an Jiaotong University, Xi’an 710049, China

## Abstract

**Motivation:**

Artificially making clinical decisions for patients with multi-morbidity has long been considered a thorny problem due to the complexity of the disease. Drug recommendations can assist doctors in automatically providing effective and safe drug combinations conducive to treatment and reducing adverse reactions. However, the existing drug recommendation works ignored two critical information. (i) Different types of medical information and their interrelationships in the patient’s visit history can be used to construct a comprehensive patient representation. (ii) Patients with similar disease characteristics and their corresponding medication information can be used as a reference for predicting drug combinations.

**Results:**

To address these limitations, we propose DAPSNet, which encodes multi-type medical codes into patient representations through code- and visit-level attention mechanisms, while integrating drug information corresponding to similar patient states to improve the performance of drug recommendation. Specifically, our DAPSNet is enlightened by the decision-making process of human doctors. Given a patient, DAPSNet first learns the importance of patient history records between diagnosis, procedure and drug in different visits, then retrieves the drug information corresponding to similar patient disease states for assisting drug combination prediction. Moreover, in the training stage, we introduce a novel information constraint loss function based on the information bottleneck principle to constrain the learned representation and enhance the robustness of DAPSNet. We evaluate the proposed DAPSNet on the public MIMIC-III dataset, our model achieves relative improvements of 1.33%, 1.20% and 2.03% in Jaccard, *F*1 and PR-AUC scores, respectively, compared to state-of-the-art methods.

**Availability and implementation:**

The source code is available at the github repository: https://github.com/andylun96/DAPSNet.

## 1 Introduction

In recent years, the widespread usage of patient Electronic Health Records (EHRs) has promoted the development of intelligent healthcare, which provides clinical decision support for doctors and improves the quality and efficiency of disease diagnosis and treatment recommendations (Li *et al.*, 2020; Ma *et al.*, 2017, 2020a, b; Nguyen *et al.*, 2021; Zhang *et al.*, 2020). Drug recommendation, as an important task, can provide effective and safe prescriptions for doctors’ reference (Cheng *et al.*, 2019; Choi *et al.*, 2016a; Li *et al.*, 2017). Existing work learns patient representations (PRs) by capturing temporal patterns of patient medical information in EHRs to accurately predict drug combinations. Such process can be carried out in two ways: (i) *Instance-based drug recommendation* (Gong *et al.*, 2021; Zhang *et al.*, 2017) that only uses the patient’s current diagnosis and procedure records for drug recommendation while ignoring the longitudinal patient history information. Therefore, the instance-based methods cannot capture the patient’s historical disease process. In order to overcome this issue, (ii) *longitudinal-based drug recommendation* (Choi *et al.*, 2016b; Shang *et al.*, 2019; Wang *et al.*, 2021; Wu *et al.*, 2022b; Yang *et al.*, 2021a, b) that models longitudinal patient history in the temporal dimension and combines learned PRs with drug representations to predict drug combinations.

However, considering the complexity of patients’ clinical treatment, the existing methods still suffer from two major challenges. **(i) Inadequate PR.** Most existing works (Choi *et al.*, 2016b; Shang *et al.*, 2019; Yang *et al.*, 2021b) only regard the diagnosis and procedure information in the patient’s historical visits as two independent views, these methods encode the medical information separately and concatenate them together to obtain the PR (Hochreiter and Schmidhuber, 1997). However, on the one hand, the obtained PR is not comprehensive, as it neither takes into account the impact of historical prescriptions on the patient’s disease state nor the relationship between medical perspectives on different dimensions of the disease trajectories. On the other hand, most deep learning methods are equipped with multi-layer neural networks to model the long-term visit dependence of patients. But more layers will lead to the increase of mutual information between the obtained PR and the input patient information, as well as the oblivion of critical information reflecting the patient’s disease state. **(ii) Inaccurate patient similarity.** The existing works (Suo *et al.*, 2017; Zhang *et al.*, 2014) exploit similarities between patients’ global representations to provide recommendations. However, patients with similar disease courses are the basis of personalized prediction, considering that in clinical practice the course of patients is often long and complex (Allam *et al.*, 2020). Therefore, compared with calculating the similarity of patient status, the similarity of patient global representation may lose or underutilize the correlation between patient disease processes to accurately match drugs and disease status.

To overcome the above challenges, we propose a deep learning model Dual Attention and Patient Similarity Network (DAPSNet) that fully utilizes the longitudinal information into PRs while integrating patient similarity to improve the performance of drug recommendation. Specifically, our DAPSNet consists of: (**i) a PR module** that utilizes code- and visit-level attention mechanisms to encode comprehensive PRs by integrating diagnosis, procedure and drug information from patients’ historical visits and current visit. (**ii) A patient retrieval module**, which constructs a patient representation memory (PM) to store the disease state representations and corresponding drug combinations of different patients, and further retrieves corresponding drug information based on the similarity of the patient’s current state with its own historical state and other patients’ historical states. (**iii) A drug recommendation module** that learns patient–drug matching by concatenating the PRs and the captured disease state similarity based drug information, then predicting the drug combinations via a sigmoid layer.

In the training stage, the DAPSNet is optimized by multiple loss functions. To reduce the information loss when learning PRs and enhance the robustness of the model, we introduce an information constrained (IC) loss function based on the information bottleneck (IB) principle (Tishby and Zaslavsky, 2015). The IC loss function aims to maximize the mutual information between PRs and labels, while minimizing the mutual information between PRs and the input patient information. Moreover, we adopt the multi-label prediction (MP) loss and drug–drug interaction (DDI) loss for guiding the model in making accurate and safe predictions. Experiments on the public MIMIC-III dataset demonstrate the effectiveness and safety of our model.

Our main contributions are summarized as follows:

- We propose DAPSNet, a novel drug recommendation model that predicts accurate and safe drug recommendation by leveraging various medical information in a patients’ history visits as well as the similarity of patients’ disease states.
- We design a novel PR module that obtains a comprehensive PR by combining the patient’s visit information encoded by code- and visit-level attention mechanisms.
- We introduce an information constraint loss to constrain the learned representation and further enhance the robustness of our model.
- We design a novel patient retrieval module that contains a PM that including all patients’ states representations and corresponding drug combinations. Furthermore, we retrieve corresponding drug information based on the patient state similarity to improve the performance of drug recommendation.
- We conduct extensive experiments on the MIMIC-III database with several state-of-the-art methods, our model outperforms the best baselines with 1.33%, 1.20%, 2.03% and 0.59% in Jaccard similarity, *F*1-score, Precision Recall Area Under Curve (PR-AUC) and Receiver Operating Characteristic Area Under Curve (ROC-AUC), respectively. The experimental results demonstrated that our proposed DAPSNet is effective, safe and robust.

## 2 Related work

According to the PR learning strategies, existing drug recommendation methods can be divided into rule-based, instance-based and longitudinal-based drug recommendation.

1. *Rule-based drug recommendation.* Rule-based drug recommendation (Almirall *et al.*, 2012; Chen *et al.*, 2016) relies on the medical guidelines summarized by clinicians, which requires a lot of medical resources and efforts. For example, Chen *et al.* (2016) designs the knowledge patterns system to recommend treatment with the patient’s medical information. However, these methods are highly limited and lack of generalization.
2. *Instance-based drug recommendation.* Instance-based drug recommendation (Gong *et al.*, 2021; Zhang *et al.*, 2017) only learns the PR from the current visit. For example, Zhang *et al.* (2017) encode the patient’s current visit with the attention mechanism and proposed a multi-instance multi-label learning framework. Gong *et al.* (2021) leverage multiple data sources to learn the embeddings of the patient–disease–medicine relations by the knowledge graph for drug recommendation. However, these methods ignore the longitudinal patient historical information.
3. *Longitudinal-based drug recommendation.* Longitudinal-based drug recommendation (Choi *et al.*, 2016b; Shang *et al.*, 2019; Wu *et al.*, 2022a, b; Yang *et al.*, 2021a, b) leverages the temporal dependencies of the longitudinal patient history and captures the sequential dependency between visits to learn the PRs. Most work uses RNN-like methods to model the longitudinal patient information. For example, Shang *et al.* (2019) adopt the memory-based networks with RNNs to model the longitudinal treatment trajectories and integrate the DDIs. Yang *et al.* (2021b) use a graph-based encoder to model the molecule information and improve the drug representation learning. Yang *et al.* (2021a) and Wu *et al.* (2022b) transform the drug recommendation task into the problem of drug change prediction. MICRON (Yang *et al.*, 2021a) is proposed to model the health condition changes by a recurrent residual learning approach, and COGNet (Wu *et al.*, 2022b) uses the copy-or-predict mechanism to model the relationship between drug changes in patients’ continuous visits.

However, few works have focused on constructing patient disease state representations that can reflect the temporal complexity of disease processes. Furthermore, due to the limitations of the model, it is difficult for existing methods to capture the patients’ long range visit dependency. This article utilizes dual view attention mechanisms to encode comprehensive PRs while integrating patient similarity to improve the performance of drug recommendation.

## 3 Materials and methods

In this section, we first define the notation and formulate the drug recommendation problem. Thereafter, we introduce the proposed DAPSNet.

### 3.1 Preliminaries and problem formulation

#### 3.1.1 Preliminaries

**Preliminaries** The longitudinal EHRs contain a variety of sequential medical codes of patients, e.g. diagnosis, procedures and drugs. Each patient can be represented as a series of clinical treatment events, taking patient *i* as an example, $X_{i}=[x_{i}{}^{\left(1\right)},x_{i}{}^{\left(2\right)},\ldots ,x_{i}{}^{\left(T_{i}\right)}]$, where i∈{1,2,···,N}, N is the number of all patients in the dataset, and $T_{i}$ denotes the total number of visits for patient *i*. We utilize a tuple $\left[d_{i}^{\left(t\right)},p_{i}^{\left(t\right)},m_{i}^{\left(t\right)}\right]$ to represent the clinical visit $x_{i}^{\left(t\right)}$ of the patient *i*, where $d_{i}{}^{\left(t\right)}\in {\left\{0,1\right\}}^{\left|D\right|},\;p_{i}{}^{\left(t\right)}\in {\left\{0,1\right\}}^{\left|P\right|}$ and $m_{i}{}^{\left(t\right)}\in {\left\{0,1\right\}}^{\left|M\right|}$ are multi-hot diagnoses, procedure and drug vectors, respectively, while D,P,M are the diagnosis, procedure and drug sets, respectively. Meanwhile, the disease state from $x_{i}{}^{\left(1\right)}$ to $x_{i}{}^{\left(t\right)}$ of patient *i* is denoted as $X_{i}{}^{1:t}$.

**EHR&DDI Graphs** We use $G_{E}=\left\{M,E_{E}\right\}$ and $G_{D}=\left\{M,E_{D}\right\}$ to denote the EHR graph and DDI graph, respectively, where M is the drug set, $E_{E}$ and $E_{D}$ are the edge set of the prescription in EHRs and known DDIs from external knowledge, respectively. We use the adjacency matrix $A_{E},A_{D}\in R^{\left|M|\times |M\right|}$ to store the edge information in $E_{E}$ and $E_{D}$. $A_{E}[i,j]=1$ means that the *i*th drug and the *j*th drug appear in the prescription in the same visit, $A_{D}[i,j]=1$ represents the adverse reaction between the *i*th drug and the *j*th drug.

#### 3.1.2 Problem formulation

In our drug recommendation task, we aim at predicting the drug combination set ${\hat{m}}^{\left(t\right)}\in {\left\{0,1\right\}}^{\left|M\right|}$ for different patient at visit *t*. Assuming that at the visit time *t* of patient *i*, given the patient disease state $X_{i}{}^{1:t-1}$, the current diagnoses and procedures code $\left[d_{i}^{\left(t\right)},p_{i}^{\left(t\right)}\right]$, EHR graph $G_{E}$ and DDI graph $G_{D}$. Our model aims to minimize the gap between the current prediction ${\hat{m}}_{i}{}^{\left(t\right)}$ and the real prescription $m_{i}{}^{\left(t\right)}\in {\left\{0,1\right\}}^{\left|M\right|}$.

The main notations used in this article are listed in Table 1.

**Table 1.** Notations used in DAPSNet

Notation	Description
$x_{i}{}^{\left(t\right)}$	The clinical visit of the patient *i* at visit *t*
$d_{i}^{\left(t\right)},p_{i}^{\left(t\right)},m_{i}^{\left(t\right)}$	The diagnosis, procedure and drug code
D,P,M	The diagnosis, procedure and drug set
$X_{i}{}^{1:t}$	The disease state process of patient *i*
$E_{*}\in R^{|*|\times \dim}$	The medical embeddings
$\alpha_{i}{}^{\left(t\right)}$	The code-level attention
$\beta_{i}{}^{\left(t\right)}$	The visit-level attention
$G_{E},G_{D}$	The EHR and DDI graph
$q_{i}{}^{\left(t\right)}$	The patient representation
${\text{sim}}_{C}$	The current similarity
${\text{sim}}_{H}^{\mathrm{Per}}$	The personal historical similarity
${\text{sim}}_{H}^{\mathrm{Pat}}$	The patient historical similarity
$C_{i}^{\left(t\right)}$	The similarity representation
$H_{\mathrm{Per}}{}_{i}^{\left(t\right)}$	The personal similarity representation
$H_{\mathrm{Pat}}{}_{i}^{\left(t\right)}$	The patient similarity representation
${\hat{m}}_{i}{}^{\left(t\right)}$	The drug predictions of the patient *i* at visit *t*
$m_{i}{}^{\left(t\right)}$	The prescription of the patient *i* at visit *t*

### 3.2 DAPSNet

**Fig. 1.**
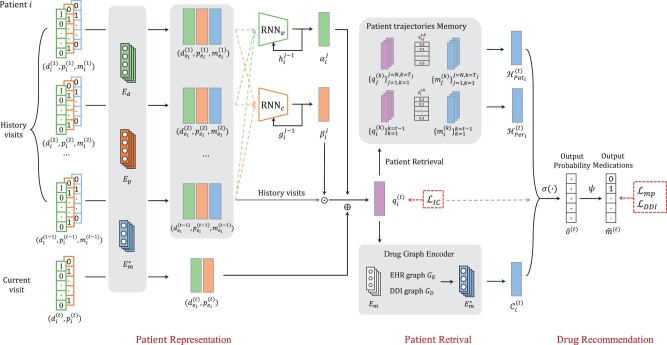
The architecture of DAPSNet. Our DAPSNet consists of a PR module, a patient retrieval module and a drug recommendation module. The PR module includes two attention mechanisms to encode the patient EHRs and yield the PRs by integrating the patient visits information. The patient retrieval module first constructs the PM and further utilizes the patient similarity to obtain additional drug information. The drug recommendation module outputs the final drug combinations

As illustrated in Figure 1, our DAPSNet consists of the following three components: (i) the **PR Module** that learns the patient disease state representation from the medical codes in the longitudinal history data; (ii) the **Patient Retrieval Module** that utilizes the patient similarity to generate additional PRs. (iii) the **drug recommendation Module** that predicts the drug combinations based on the concatenated patients’ representations. Each component of the DAPSNet is detailed below in turn.

**(i) PR Module**

In the longitudinal EHRs, each medical code plays an important role in PR. The diagnosis, procedure and drug information reflect the patient’s health status, treatment process and historical prescription, respectively. In order to make full use of these medical information, we design a patient encoder, which includes the designed embedding tables of different medical codes. We first embed the disease state from $x_{i}{}^{\left(1\right)}$ to $x_{i}{}^{\left(t-1\right)}$ of patient *i*, $X_{i}{}^{1:t-1}=[d_{i}^{\left(1\right)},p_{i}^{\left(1\right)},m_{i}^{\left(1\right)}],\ldots ,[d_{i}^{\left(t-1\right)},p_{i}^{\left(t-1\right)},m_{i}^{\left(t-1\right)}]$ and the current medical code $\left[d_{i}^{\left(t\right)},p_{i}^{\left(t\right)}\right]$ into the embedding space:

(1) $$\begin{array}{c} d_{e_{i}}^{\left(j\right)}=d_{i}^{\left(j\right)}E_{d},j\in \{1,2,\ldots ,t\}\\ p_{e_{i}}^{\left(j\right)}=p_{i}^{\left(j\right)}E_{p},j\in \{1,2,\ldots ,t\}\\ m_{e_{i}}^{\left(j\right)}=m_{i}^{\left(j\right)}E_{m}^{*},j\in \{1,2,\ldots ,t-1\}, \end{array}$$

where j∈{1,2,…,t} indicates the index of visit while the medical code are diagnosis and procedure information, j∈{1,2,…,t−1} when the medical code is drug information. $E_{d}\in R^{|D|\times \dim},E_{p}\in R^{|P|\times \dim},E_{m}^{*}\in R^{|M|\times \dim}$ are the embedding tables of diagnosis, procedure and drug, respectively ($E_{m}^{*}$ will be explained in the next section), and $d_{e_{i}}^{\left(j\right)},p_{e_{i}}^{\left(j\right)},m_{e_{i}}^{\left(j\right)}$ are embedding vectors of the diagnoses, procedures and drug of patient *i* at visit *j*, respectively. Thus, the disease process of patient $i\;X_{i}{}^{1:t-1}$ can be represented as $e_{i}{}^{1:t-1}=[d_{e_{i}}^{\left(1\right)},p_{e_{i}}^{\left(1\right)},m_{e_{i}}^{\left(1\right)}],\ldots ,[d_{e_{i}}^{\left(t-1\right)},p_{e_{i}}^{\left(t-1\right)},m_{e_{i}}^{\left(t-1\right)}]$, the current medical code can be represented as the embedded vector $e_{i}{}^{t}=[d_{e_{i}}^{\left(t\right)},p_{e_{i}}^{\left(t\right)}]$.

**Drug Graph encoder**

There are two kinds of graph structure information in the EHR data and external knowledge. The EHR graph contains the information that some drugs are prescribed at the same time to improve the curative effect, and the DDI graph contains the information that some drugs have adverse reactions and cannot be used at the same time. Inspired by the GAMENet (Shang *et al.*, 2019) using the Graph Convolutional Network (GCN) (Kipf and Welling, 2016) to encode the drug representation. In order to recommend effective and safe drug combinations, we encode the EHR graph $G_{E}$ and DDI graph $G_{D}$ to obtain the drug representation.

Given the input drug embedding table $E_{m}\in R^{|M|\times \dim}$ and the drug adjacency matrix $A_{*}\in R^{\left|M|\times |M\right|}$, we use the GCN layer to obtain the drug representations as follows:

(2) $$\text{GCN}(E_{m},A_{*})=\sigma ({\hat{D}}^{-\frac{1}{2}}\hat{A_{*}}{\hat{D}}^{-\frac{1}{2}}E_{m}W),$$

where $\hat{D}$ is a diagonal matrix of $\hat{A_{*}}$ (e.g. ${\hat{D}}_{ii}=\Sigma_{j}A_{*}{}_{ij}$), $\hat{A_{*}}=A_{*}+I,\;I$ is the identity matrix.

Then, we use a two-layer GCN to model the improved embeddings on each graph. We model the co-occurrence relations and DDIs based on the EHRs and DDIs adjacency matrix *A_E_* and *A_D_* separately.

(3) $$G_{e}=\text{GCN}(\text{tanh}(\text{GCN}(E_{m},A_{E}))W_{e},A_{E}),$$

(4) $$G_{d}=\text{GCN}(\text{tanh}(\text{GCN}(E_{m},A_{D}))W_{d},A_{D}),$$

where *W_e_* and *W_d_* are the hidden learnable parameter matrices and *G_e_* and *G_d_* are the generated drug relation representations.

Finally, we fuze two generated relation representations *G_e_*, *G_d_* together to obtain the final drug representation $E_{m}^{*}$,

(5) $$E_{m}^{*}=G_{e}+\delta G_{d},$$

where *δ* is the learnable parameter to fuze different relation graphs.

**Attention mechanisms**

Inspired by the AMANet (He *et al.*, 2020), which learns the intra-view interaction and inter-view interaction for dual asynchronous sequential learning through the self- and inter-attention mechanisms, respectively. In the EHRs data, we treat the diagnosis, procedure and drug in the disease process as three sequential views. Based on the above embedding process, in order to select the relative important visits in the disease process and the critical medical code in each visit, we design two different attention mechanisms, namely, the code-level attention and visit-level attention, which give different weights to different medical codes and different visit records in the patient disease process.

Firstly, in order to select the critical medical code in each visit (e.g. $e_{i}{}^{\left(j\right)}=[d_{e_{i}}^{\left(j\right)},p_{e_{i}}^{\left(j\right)},m_{e_{i}}^{\left(j\right)}]$), we design the code-level attention mechanism to obtain the weight corresponding to different codes in each visit $\alpha_{i}^{\left(1\right)},\alpha_{i}^{\left(2\right)},\ldots ,\alpha_{i}^{\left(t-1\right)}$, which can be measured by:

(6) $$g_{i}^{\left(j\right)}={\text{RNN}}_{C}(g_{i}^{\left(j-1\right)},e_{i}^{j}),$$

(7) $$\alpha_{i}^{\left(j\right)}=\tanh(W_{g}g_{i}^{\left(j\right)}+b_{g}),$$

where *W_g_* and *b_g_* are the learnable parameters.

Moreover, in order to select the relative important visits in the patient disease process $e_{i}{}^{1:t-1}$, we design the visit-level attention to obtain weights corresponding to each visit $\beta_{i}^{\left(1\right)},\beta_{i}^{\left(2\right)},\ldots ,\beta_{i}^{\left(t-1\right)}$, which can be measured by:

(8) $$h_{i}^{\left(j\right)}={\text{RNN}}_{V}\left(h_{i}^{\left(j-1\right)},e_{i}^{j}\right),$$

(9) $$\beta_{i}^{\left(j\right)}=\frac{W_{h}h_{i}^{\left(j\right)}}{\sum_{j=1}^{t-1}W_{h}h_{i}^{\left(j\right)}},$$

where *W_h_* is the learnable parameter.

Therefore, we can select the critical medical code elements in each visit and the relatively important visit in the patient disease process to jointly obtain the final PR $q_{i}^{\left(t\right)}$ by combining the above two attention mechanisms with the patient’s disease process and the current medical code. The calculation process is as follows:

(10) $$q_{i}^{\left(t\right)}=e_{i}^{t}+\sum_{j=1}^{t-1}\beta_{i}^{\left(j\right)}\cdot \left(\alpha_{i}^{\left(j\right)}⊙e_{i}^{j}\right),$$

where $q_{i}^{\left(t\right)}$ is the representation of the patient *i* with *t* times visit, the embedded vector $e_{i}{}^{t}=[d_{e_{i}}^{\left(t\right)},p_{e_{i}}^{\left(t\right)}]$ is the current medical code and ⊙ is the element-wise multiplication.

The PR module of our model is composed of embedded layers of different medical codes and two different attention mechanisms. Compared with the previous work, DAPSNet can learn a more comprehensive patient disease state representation in the longitudinal patient EHRs data, which is helpful for the subsequent patient retrieval module to measure the similarity accurately.

**(ii) Patient Retrieval Module**

We record each visit of N patients in the EHRs as

$\left\{X_{1}{}^{1:T_{1}},\ldots ,X_{N}{}^{1:T_{N}}\right\}$ . Our patient retrieval module first constructs a PM to store the disease state representations and the corresponding drug combinations of different patients, and further calculates the similarity information between the PRs from PM to retrieve the corresponding drug information. The retrieval process can be separated into the following three steps.

First, we build a PM to store the patient disease state representations ${\left\{q_{j}^{\left(k\right)}\right\}}_{j=1,k=1}^{j=N,k=T_{j}}$ learned from the PR module and the corresponding drug combinations ${\left\{m_{j}^{\left(k\right)}\right\}}_{j=1,k=1}^{j=N,k=T_{j}}$ in each visit.

- *Current Similarity*: Next, we calculate the similarity between the patient’s current representation $q_{i}^{\left(t\right)}$ and the drug representation $E_{m}^{*}$, which we record as the Current Similarity ${\text{sim}}_{C}$. Using the current similarity, we can directly retrieve the Current Similarity drug information $C_{i}^{\left(t\right)}$,
  (11) $$C_{i}^{\left(t\right)}=E_{m}^{*}\cdot {\text{sim}}_{C}(q_{i}^{\left(t\right)},E_{m}^{*}),$$
  (12) $${\text{sim}}_{C}(q_{i}^{\left(t\right)},E_{m}^{*})=\text{Softmax}(E_{m}^{*}q_{i}^{\left(t\right)}),$$
  where the Current Similarity ${\text{sim}}_{C}(\cdot ,\cdot )$ calculates the similarity matrix between the PR $q_{i}^{\left(t\right)}$ and the drug representation $E_{m}^{*}$, then, we use Softmax function to normalize the weight matrix.
- *Historical Similarity*: Due to the temporal complexity of patient disease processes, take patient *i* as example, the disease state representation at visit *t* of the target patient *i*: $q_{i}^{\left(t\right)}$ may have high similarity to (i) his/her own historical visits ${\left\{q_{i}^{\left(k\right)}\right\}}_{k=1}^{k=t-1}$ and (ii) the historical visits of other patients ${\left\{q_{j}^{\left(k\right)}\right\}}_{j=1,k=1}^{j=N(j\neq i),k=T_{j}}$.

Therefore, we first calculate the similarity between the representation of the patient’s current visit $q_{i}^{\left(t\right)}$ and the representations of each visit in his/her own history ${\left\{q_{i}^{\left(k\right)}\right\}}_{k=1}^{k=t-1}$. We record as the Personal Historical Similarity ${\text{sim}}_{H}^{\mathrm{Per}}$. We can retrieve the Personal Similarity drug information $H_{\mathrm{Per}}{}_{i}^{\left(t\right)}$ with the following steps,

(13) $$s_{i}{}^{\left(t,k\right)}={\text{sim}}_{H}^{\mathrm{Per}}(q_{i}^{\left(t\right)},q_{i}^{\left(k\right)})=\frac{q_{i}^{\left(t\right)}\cdot q_{i}^{\left(k\right)}}{\left|q_{i}^{\left(t\right)}\right|\cdot \left|q_{i}^{\left(k\right)}\right|},$$

(14) $$H_{\mathrm{Per}}{}_{i}^{\left(t\right)}=E_{m}^{*}\cdot {\text{sim}}_{H}^{\mathrm{Per}}(s_{i}{}^{\left(t,k\right)},{\left\{m_{i}^{\left(k\right)}\right\}}_{k=1}^{k=t-1}),$$

where $s_{i}{}^{\left(t,k\right)}$ denotes the similarity between the representation $q_{i}{}^{\left(t\right)}$ and ${\left\{q_{i}^{\left(k\right)}\right\}}_{k=1}^{k=t-1}$, then, we use the sequence similarity $s_{i}{}^{\left(t,k\right)}$ as attention weights to generate history drugs distribution by weighted sum of the corresponding drugs ${\left\{m_{i}^{\left(k\right)}\right\}}_{k=1}^{k=t-1}$. Finally, with further retrieving information from $E_{m}^{*}$ using ${\text{sim}}_{H}^{\mathrm{Per}}$, we can get the Personal Similarity drug information $H_{\mathrm{Per}}{}_{i}^{\left(t\right)}$.

Similar to measure the similarity with the patient’s own historical visits, we calculate the similarity between the target patient’s representation $q_{i}^{\left(t\right)}$ and the representation of different patients at each visit, we record as the Patient Historical Similarity ${\text{sim}}_{H}^{\mathrm{Pat}}$. We can retrieve the Patient Similarity drug information $H_{\mathrm{Pat}}{}_{i}^{\left(t\right)}$ with the following steps,

(15) $$s_{i,j}{}^{\left(t,k\right)}={\text{sim}}_{H}^{\mathrm{Pat}}(q_{i}^{\left(t\right)},q_{j}^{\left(k\right)})=\frac{q_{i}^{\left(t\right)}\cdot q_{j}^{\left(k\right)}}{\left|q_{i}^{\left(t\right)}\right|\cdot \left|q_{j}^{\left(k\right)}\right|},$$

(16) $$H_{\mathrm{Pat}}{}_{i}^{\left(t\right)}=E_{m}^{*}\cdot {\text{sim}}_{H}^{\mathrm{Pat}}(s_{i,j}{}^{\left(t,k\right)},{\left\{m_{j}^{\left(k\right)}\right\}}_{j=1,k=1}^{j=N,k=T_{j}}),$$

where $s_{i,j}{}^{\left(t,k\right)}$ denotes the similarity between the representation $q_{i}{}^{\left(t\right)}$ and ${\left\{q_{j}^{\left(k\right)}\right\}}_{j=1,k=1}^{j=N(j\neq i),k=T_{j}}$, similar with the calculation process of Personal Historical Similarity, we can get the Patient Similarity drug information $H_{\mathrm{Pat}}{}_{i}^{\left(t\right)}$. Meanwhile, to avoid excessive computational complexity, we only consider the top *n* sequences.

Finally, we concatenate two drug information with different similarities $\left[H_{\mathrm{Per}}{}_{i}^{\left(t\right)},H_{\mathrm{Pat}}{}_{i}^{\left(t\right)}\right]$ to get our Historical Similarity representation $H_{i}^{\left(t\right)}$.

**(iii) Drug recommendation Module**

After retrieving the Current Similarity representation $C_{i}^{\left(t\right)}$, the Historical Similarity representation $H_{i}^{\left(t\right)}$ and the patient current representation $q_{i}^{\left(t\right)}$, we need to find the drug combinations that are most relevant to the patient’s current disease state. Following previous work (Shang *et al.*, 2019), we use a patient–drugs matching function, the MP $o_{i}^{\left(t\right)}$ can be obtained as:

(17) $$o_{i}^{\left(t\right)}=\text{sigmoid}(\text{concat}[q_{i}^{\left(t\right)},C_{i}^{\left(t\right)},H_{i}^{\left(t\right)}]),$$

where $o_{i}^{\left(t\right)}\in R^{\left|M\right|}$ denotes the final matching scores for the patient *i*. By comparing the matching scores $o_{i}^{\left(t\right)}$ to a pre-defined threshold parameter *ψ*, we can obtain the final drug combinations ${\hat{m}}_{i}{}^{\left(t\right)}\in R^{\left|M\right|}$ predicted by our model.

**(iv) Loss Function**

Our DAPSNet is trained with three loss functions: (i) a *DDI Loss* for explicitly constraining the DDI rate in the drug combinations prediction, (ii) a *MP Loss* for accurately predicting the drug combinations and (iii) an *Information Constraint Loss* to enhance the robustness of the model with utilizing the IB principle. We simultaneously optimize the learnable parameters during the training process.

- *DDI Loss*: For the drug combinations, we want to achieve a lower DDI rate, which will reduce adverse reactions and realize the prediction of safe drug recommendation. Based on the DDI adjacency matrix *A_D_*, we design the DDI loss for a single visit $o_{i}^{\left(t\right)}$ as:
  (18) $$L_{\mathrm{DDI}}=\sum_{i=1}^{\left|M\right|}\sum_{j=1}^{\left|M\right|}A_{D}{}_{i,j}\cdot o_{i}^{\left(t\right)}\cdot o_{j}^{\left(t\right)}.$$
  During the training, the model will conduct back propagation according to the average DDI loss of all visits.
- *MP Loss*: We consider the drug recommendation as a multi-label binary classification task, and use two common multi-label loss functions. The first one is Multi-Label Margin (MLM) loss (Ji and Ye, 2009), which is popular in existing drug recommendation works, such as GAMENet (Shang *et al.*, 2019), SafeDrug (Yang *et al.*, 2021b) and COGNet (Wu *et al.*, 2022b). The MLM loss ensures the predicted probability of ground truth labels has at least 1 margin larger than others, which can be mathematically described as:
  (19) $$L_{\mathrm{multi}}=\sum_{i,j:m_{i}^{\left(t\right)}=1,m_{j}^{\left(t\right)}=0}\frac{\max(0,1-({\hat{o}}_{i}^{\left(t\right)}-{\hat{o}}_{j}^{\left(t\right)}))}{\left|M\right|}.$$
  The second one is the Binary Cross-Entropy (BCE) loss, which can be formulated as:
  (20) $$L_{\mathrm{bce}}=-\sum_{i=1}^{\left|M\right|}[m_{i}{}^{\left(t\right)}\;\text{log}({\hat{o}}_{i}{}^{\left(t\right)})+(1-m_{i}{}^{\left(t\right)})\;\text{log}(1-{\hat{o}}_{i}{}^{\left(t\right)})].$$
  The MP loss is formulated by combining the MLM loss and BCE loss with a balance hyper-parameter *μ*:
  (21) $$L_{mp}=(1-\mu )L_{\mathrm{multi}}+\mu L_{\mathrm{bce}}.$$
- *Information Constraint Loss*:
  In order to obtain a compact and comprehensive PR, we extend the IB principle to the drug recommendation task. Specifically, in this work, we encourage to minimize the mutual information between the latent PRs *q* and the input medical codes *X* while maximizing the mutual information between the latent representations *q* and the drug labels *m*,
  (22) minI(X;q)−φI(q;m).
  According to the variational approximation of the IB (Alemi *et al.*, 2016), we can get the lower bound of I(q;m), thus, the latter term of the objective function Equation (21) is equal to the BCE loss mentioned above.
  According to the definition of the mutual information, I(X;q)=H(q)−H(q|X), during the training, the model will get a definite latent PR *q* with given medical codes input *X*, so the conditional entropy of *q* given *X*: H(q|X)=0, Therefore, minimizing the mutual information I(X;q) can be estimated to minimize the entropy of *q*, *H*(*q*). The latent PRs *q* consists multiple visits *q^t^*, given a real valued positive definite kernel *κ* and the Gram matrix K, where $K_{i,j}=\kappa (q^{i},q^{j})$, where *q^i^* and *q^j^* are the representations of the *i*-th and *j*-th samples in a batch, respectively. We use a matrix-based analogue to R$\overset{’}{e}$nyi’s *α*-entropy to approximate calculate *H*(*q*) (Yu *et al.*, 2021),
  (23) $$\begin{array}{c} H(q)=H_{\alpha }(A)=\frac{1}{1-\alpha }\;{\text{log}}_{2}(\text{tr}(A^{\alpha }))\\ =\frac{1}{1-\alpha }\;{\text{log}}_{2}\left(\sum_{i=1}^{n}\lambda_{i}{\left(A\right)}^{\alpha }\right), \end{array}$$
  where α∈(0,1)∩(1,+∞), A is the normalized version of $K,\;A=\frac{K}{\text{tr}(K)},\;\lambda_{i}(A)$ denotes the *i*-th eigenvalue of A. To simplify the formulation of IB, we define the last item of Equation (23) as the information constraint loss $L_{IC}$.
- *Overall Loss Function*: During the training process, the overall loss function L is obtained by combining the three loss functions through the weighted sum to optimize the drug recommendation network,
  (24) $$L=\lambda_{1}L_{\mathrm{DDI}}+\lambda_{2}L_{mp}+\lambda_{3}L_{IC},$$
  where *λ*_1_, *λ*_2_ and *λ*_3_ are the weights for different loss functions.

**(v) Algorithm**

Our training algorithm is detailed in Algorithm 1.

Algorithm 1
One training epoch of DAPSNet

**Require:** Training set ${\left\{(d_{i},p_{i},m_{i})\right\}}_{i=1}^{N}$, EHRs and DDIs knowledge matrices $A_{E},A_{D}$, hyper-parameters *δ*, *μ*, *λ*_1_, *λ*_2_ and *λ*_3_ and the threshold *ψ*;

1: Initialize the parameters: *E_d_*, *E_p_*, *E_m_*, ${\text{RNN}}_{c},\;{\text{RNN}}_{v}$, *W_e_* and *W_d_*;

2: Generate the drug representation $E_{m}^{*}$ according to Equations (2)–(5);

3: **for** patient i:=1 to *N* **do**

4: Read the patient i′s history $\left[d^{\left(1\right)},d^{\left(2\right)},\ldots ,d^{\left(T\right)}],\;[p^{\left(1\right)},p^{\left(2\right)},\ldots ,p^{\left(T\right)}],\;[m^{\left(1\right)},m^{\left(2\right)},\ldots ,m^{\left(T\right)}\right]$;

5:  **for** history visit *t*:= 1 to *T* **do**

6:  Read the historical diagnoses, procedures and drugs of the patient at the *t*-th visit $\left[d^{\left(1\right)},\ldots ,d^{\left(t\right)}],\;[p^{\left(1\right)},\ldots ,p^{\left(t\right)}\right]$ and $\left[m^{\left(1\right)},\ldots ,m^{\left(t-1\right)}\right]$;

7:   Generate embeddings $d_{e_{i}}^{\left(j\right)},\;p_{e_{i}}^{\left(j\right)}\;(j\leq t)$ and $m_{e_{i}}^{\left(j\right)},(j\leq t-1)$ by Equation (2);

8:   Generate attention vector $\alpha_{i}{}^{\left(j\right)}$ and $\beta_{i}{}^{\left(j\right)}$ by Equations (6)–(9);

9:   Generate patient representation $q_{i}{}^{\left(t\right)}$ by Equation (10);

10:   Generate Current Similarity representation $C_{i}^{\left(t\right)}$ by Equations (11) and (12);

11:   Generate Historical Similarity representation $H_{i}^{\left(t\right)}$ by Equations (13)–(16);

12:   Generate mapping representation $o_{i}^{\left(t\right)}$ by Equation (17);

13:   Generate the output multi-hot drug vector ${\hat{m}}_{i}{}^{\left(t\right)}$ by comparing the matching scores $o_{i}{}^{\left(t\right)}$ with *ψ*.

14:  **end for**

15:  Generate and accumulate $L_{\mathrm{DDI}},L_{mp},L_{IC}$ in Equations (18) and (23), respectively;

16: **end for**

17: Optimize the combined loss L in Equation (24);

## 4 Experiments setup

### 4.1 Dataset

We evaluate the effectiveness and safety of the proposed DAPSNet and baselines on the public MIMIC-III database (Johnson *et al.*, 2016). Our dataset is processed according to the protocol proposed by PhysioNet (Goldberger *et al.*, 2000). Following the data-preprocessing in the previous work (Shang *et al.*, 2019; Yang *et al.*, 2021b), we choose the drugs within the first 24 h and only keep the patients with more than one visit in our dataset. The diagnosis and procedure data are coded by the ninth version of International Classification of Diseases (http://www.icd9data.com/). We extract DDI information of the top-40 most common types from TWOSIDES (Tatonetti *et al.*, 2012), where the drugs are presented by ATC third level codes (https://www.whocc.no/atc/structure_and_principles/). In order to integrate the DDI data and compute the DDI score, we transform the NDC codes to the same ATC third level codes.

We stratified patients with different visit times in the dataset. The statistics of the processed MIMIC-III dataset are summarized in Table 2.

**Table 2.** Statistics of the dataset

Item	Number
# patients	6350
# clinical events	14 995
# diagnoses	1958
# procedures	1430
# drugs (ATC third)	132
Avg./max # of visits	2.37/29
Avg./max # of diagnoses per visit	10.51/128
Avg./max # of procedures per visit	3.84/50
Avg./max # of drugs per visit	11.18/64
# patients visited twice	4700
# patients visited three times	949
# patients visited four times	373
# patients visited five times	170
# patients visited more than five times	158

### 4.2 Baselines

We evaluate our model by comparing it with the following baselines including Logistic Regression (LR),ECC, RETAIN (Choi *et al.*, 2016b), LEAP (Zhang *et al.*, 2017), DMNC (Le *et al.*, 2018), GAMENet (Shang *et al.*, 2019), SafeDrug (Yang *et al.*, 2021b), MICRON (Yang *et al.*, 2021a) and COGNet (Wu *et al.*, 2022b).

- **LR** is a standard logistic regularization.
- **ECC** uses multiple SVM classifiers to make MP;
- **RETAIN** (Choi *et al.*, 2016b) uses a two-level RNN neural attention model to improve the sequence prediction.
- **LEAP** (Zhang *et al.*, 2017) is an instance-based method, which treats drug recommendation as a sentence generation task.
- **DMNC** (Le *et al.*, 2018) uses a memory augmented neural network to recommend on a differentiable neural network.
- **GAMENet** (Shang *et al.*, 2019) adopts memory augmented neural networks and stores historical prescriptions for prediction.
- **SafeDrug** (Yang *et al.*, 2021b) captures the molecule structure information with the global and local encoders.
- **MICRON** (Yang *et al.*, 2021a) proposes an recurrent residual learning model to predict the drug change.
- **COGNet** (Wu *et al.*, 2022b) utilizes a copy-or-predict mechanism to generate the drug combinations.

### 4.3 Evaluation metrics

We use DDI rate, Jaccard similarity score (Jaccard) (Niwattanakul *et al.*, 2013), Average *F*1 score (*F*1), PR-AUC (Davis and Goadrich, 2006) and ROC-AUC to measure the safety, accuracy and effectiveness of prediction.

### 4.4 Implementation details

Following the previous drug recommendation work (Shang *et al.*, 2019; Wu *et al.*, 2022b; Yang *et al.*, 2021b), we divided the dataset into training, validation and test set as $\frac{2}{3}:\frac{1}{6}:\frac{1}{6}$. Our method is implemented by PyTorch (https://pytorch.org) 1.6.0 based on python 3.7.5, tested on an Intel Xeon CPUs with two NVIDIA 2080Ti GPUs. We choose the optimal hyper-parameters in our model based on the validation set, the dimension size is set to 64 and the threshold *δ* is set to 0.5. The weight *μ*, *λ*_1_, *λ*_2_ and *λ*_3_ in our overall loss are set to 0.05, 0.2, 0.75 and 0.05, respectively. We use a $2\times 10^{-4}$ learning rate to train our model within 50 epochs. Our model is optimized by the Adam optimizer (Kingma and Ba, 2014). All the baselines are trained and implemented with the optimized parameters from the references.

## 5 Results and discussion

### 5.1 Results

#### 5.1.1 Performance comparison

Table 3 demonstrates the experiment results of the proposed DAPSNet and baselines. We conduct 10 rounds of tests for all the models and report their metric scores’ mean and standard deviation. Overall, our proposed model DAPSNet outperforms all baselines in terms of 1.33%, 1.20%, 2.03% and 0.59% improvement in Jaccard similarity, *F*1-score, PR-AUC and ROC-AUC, respectively. Compared with the DL-models that utilize longitudinal patient information (e.g. RETAIN, DMNC, GAMENet, SafeDrug, MICRON and COGNet), the instance-based models (e.g. LR, ECC and LEAP) that only consider the current visit shows poor results in accuracy prediction. At the same time, the DDI rate of the predicted drug combinations is similar to the MIMIC-III dataset itself (average DDI: 0.08379).

**Table 3.** Performance comparison on MIMIC-III

Model	DDI	Jaccard	*F*1	PR-AUC	ROC-AUC	Avg. # of drugs
ECC	0.0849 ± 0.0018	0.4996 ± 0.0049	0.6569 ± 0.0044	0.6844 ± 0.0038	0.9281 ± 0.0014	18.0722 ± 0.1914
RETAIN	0.0835 ± 0.0020	0.4887 ± 0.0028	0.6481 ± 0.0027	0.7556 ± 0.0033	0.9257 ± 0.0011	20.4051 ± 0.2832
LEAP	0.0731 ± 0.0008	0.4521 ± 0.0024	0.6138 ± 0.0026	0.6549 ± 0.0033	0.9172 ± 0.0014	18.7138 ± 0.0666
DMNC	0.0842 ± 0.0011	0.4864 ± 0.0025	0.6529 ± 0.0030	0.7580 ± 0.0039	0.9226 ± 0.0043	20.0000 ± 0.0000
GAMENet	0.0864 ± 0.0006	0.5067 ± 0.0025	0.6626 ± 0.0025	0.7631 ± 0.0030	0.9288 ± 0.0064	27.2145 ± 0.1141
SafeDrug	**0.0589 **±** **0.0005	0.5213 ± 0.0030	0.6748 ± 0.0027	0.7647 ± 0.0025	0.9344 ± 0.0009	19.9178 ± 0.1604
MICRON	0.0641 ± 0.0007	0.5100 ± 0.0033	0.6654 ± 0.0031	0.7687 ± 0.0026	0.9337 ± 0.0011	17.7267 ± 0.2172
COGNet	0.0852 ± 0.0005	0.5336 ± 0.0011	0.6869 ± 0.0010	0.7739 ± 0.0009	0.9396 ± 0.0004	28.0903 ± 0.0950
DAPSNet	0.0657 ± 0.0010	**0.5469 **±** **0.0025	**0.6989 **±** **0.0013	**0.7942 **±** **0.0024	**0.9465 **±** **0.0006	21.0715 ± 0.2104

Note: The best results in each column are bolded.

In detail, RETAIN and DMNC only encode the patient’s historical information and do not introduce external knowledge into the models. In contrast, GAMENet improves the model’s performance by encoding the drug embedding of the external graph structure and constructing the patient memory bank, but it provides a high DDI rate. SafeDrug models the molecular graph in drug encoding and introduces the DDI controllable loss function, resulting in a further performance improvement and ensuring the lowest DDI rate among SOTA methods. Following the parameter chosen in SafeDrug, we set the DDI threshold in SafeDrug to 0.06. Different from the above models, MICRON and COGNet noticed that there is a correlation between drug combinations in two consecutive visits. MICRON uses the recurrent residual method to predict the unchanged drugs. Considering the correlation between drugs, COGNet introduces a copy-or-predict mechanism to determine whether historical prescriptions are still relevant, which further improves the performance but maintains a high DDI rate because of no DDI constraint. Compared with the above SOTA model, our DAPSNet achieves higher prediction accuracy under DDI constraints. Experimental results show that our model can balance the accuracy and safety of prediction through the PR and patient retrieval module and further predict safer and more effective drug combinations than the existing methods.

#### 5.1.2 Historical information utilization

**Fig. 2.**
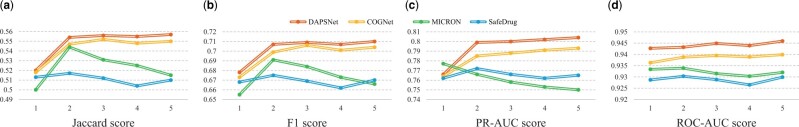
The effect of number of visits for various models

To further explore the ability of model DAPSNet to capture the medical information in historical visits, we conduct experiments on the performance of different models to investigate the impact of the number of visits in the dataset. According to the dataset statistics in Table 2, in our dataset, the average number of visits for different patients is 2.37. The proportion of patients with more than five visits in the dataset does not exceed 10%. So, we stratify the datasets based on different number of visits to study its impact on the performance of different models. As a comparison, here, we choose the recent SafeDrug, MICRON and COGNet as stronger baselines. The comparison results of various methods on different number of visits are in Figure 2. The horizontal axis represents the patients visit times and the vertical axis represents the values of the different evaluation metrics. The results show that DAPSNet almost achieves the best performance with different visit times. With the increase in visits, the performance of DAPSNet and COGNet has been further improved, showing that both models effectively use patient historical information. On the contrary, the performance of SafeDrug using RNN to model the patient history decreased, but the overall performance remains unchanged. The performance of MICRON shows a decreasing trend under different visit times, because the drug change measurement mechanism will lead to error accumulation and MICRON only predicts the unchanged part. In conclusion, we can see that our DAPSNet can stably recommend safe and effective drug combinations with more visits while comparing with other models.

### 5.2 DAPSNet components analysis

#### 5.2.1 Model ablation study

In this section, we verify the effectiveness of each module in DAPSNet. Specifically, we design the ablation studies on our dataset and test on the following variants:

- DAPSNet w/o D: we remove the diagnoses information in each visit.
- DAPSNet w/o P: we remove the procedures information in each visit.
- DAPSNet w/o M: we remove the medications information in each visit.
- DAPSNet w/o α: we remove the code-level attention mechanism in PR module.
- DAPSNet w/o β: we remove the visit-level attention mechanism in PR module.
- DAPSNet w/o α,β: we remove both the code- and visit-level attention mechanisms.
- DAPSNet $w/o\;G_{\mathrm{DDI}}$: we remove the DDI graph in encoding the drug representation.
- DAPSNet $w/o\;G_{\mathrm{EHR}}$: we remove the EHR graph in encoding the drug representation.
- DAPSNet $w/o\;G_{\mathrm{Drug}}$: we remove the EHR and DDI graphs in encoding the drug representation.
- DAPSNet $w/o\;H_{\mathrm{Pat}}{}_{i}$: we remove the set of patient historical similarity representations.
- DAPSNet $w/o\;H_{i}$: we remove the set of similarity representations.
- DAPSNet $w/o\;L_{\mathrm{DDI}}$: we remove the DDI loss function.
- DAPSNet $w/o\;L_{IC}$: we remove the Information Constraint loss function.

Table 4 shows the results for the different variants of DAPSNet. By comparing the ablation results, DAPSNet w/o D, DAPSNet w/o P and DAPSNet w/o M yield poor results among all ablation models, which suggest that the medical information, diagnosis, procedure and medication information, play an important role in the drug recommendation. DAPSNet w/o α, DAPSNet w/o β and DAPSNet w/o α,β indicate that the code- and visit-level attention mechanisms in PR learning module bring an improvement to the recommendation performance. DAPSNet $w/o\;G_{\mathrm{DDI}}$, DAPSNet $w/o\;G_{\mathrm{EHR}}$ and DAPSNet $w/o\;G_{\mathrm{Drug}}$ illustrate that the DDI graph and EHR graph in the drug graph encoding module can not only learn a more comprehensive drug representation, but also improve the performance of the model. Further, we compare the DAPSNet $w/o\;H_{\mathrm{Pat}}{}_{i}$ and DAPSNet $w/o\;H_{i}$ with our model, the result illustrates that the patient historical similarity and the personal historical similarity improve the accuracy and completeness of the patient retrieval module, thereby improving model performance. The results of DAPSNet $w/o\;L_{IC}$ indicate that IB principle constrains the learned PRs and has contributions to the final result. Compared to the variant without DDI loss $L_{\mathrm{DDI}}$, we found that DAPSNet $w/o\;L_{\mathrm{DDI}}$ has better results in some indicators(e.g. Jaccard, *F*1, PR-AUC and ROC-AUC), but the DDI rate is much higher than DAPSNet. Without the constraint of DDI loss function, DAPSNet $w/o\;L_{\mathrm{DDI}}$ has a similar DDI rate with the MIMIC-III dataset itself and the average number of recommended drugs has increased, which suggests that our DAPSNet mimics the behavior of physicians and provides better performance in prescribing drug combinations. Overall, comparing with all variants, DAPSNet achieves more balanced and accurate result in drug recommendation.

**Table 4.** Ablation study for different components of DAPSNet on MIMIC-III

Model	DDI	Jaccard	*F*1	PR-AUC	ROC-AUC	Avg. # of drugs
DAPSNet w/o D	0.0590 ± 0.0007	0.5132 ± 0.0028	0.6687 ± 0.0025	0.7664 ± 0.0031	0.9369 ± 0.0016	18.9669 ± 0.1606
DAPSNet w/o P	0.0614 ± 0.0011	0.5235 ± 0.0031	0.6794 ± 0.0028	0.7792 ± 0.0040	0.9422 ± 0.0018	20.3780 ± 0.1406
DAPSNet w/o M	0.0647 ± 0.0014	0.5363 ± 0.0019	0.6889 ± 0.0017	0.7850 ± 0.0033	0.9431 ± 0.0009	19.9799 ± 0.1670
DAPSNet w/o α	0.0674 ± 0.0013	0.5386 ± 0.0020	0.6894 ± 0.0024	0.7840 ± 0.0026	0.9434 ± 0.0006	20.2564 ± 0.1680
DAPSNet w/o β	0.0676 ± 0.0013	0.5373 ± 0.0026	0.6897 ± 0.0028	0.7837 ± 0.0027	0.9427 ± 0.0008	20.3225 ± 0.1950
DAPSNet w/o α,β	0.0667 ± 0.0014	0.5204 ± 0.0033	0.6772 ± 0.0022	0.7712 ± 0.0025	0.9392 ± 0.0018	20.3252 ± 0.2419
DAPSNet $w/o\;G_{\mathrm{DDI}}$	0.0684 ± 0.0011	0.5435 ± 0.0028	0.6962 ± 0.0025	0.7932 ± 0.0033	0.9436 ± 0.0006	19.8746 ± 0.1534
DAPSNet $w/o\;G_{\mathrm{EHR}}$	0.0624 ± 0.0009	0.5425 ± 0.0027	0.6956 ± 0.0024	0.7936 ± 0.0031	0.9434 ± 0.006	22.4862 ± 0.1684
DAPSNet $w/o\;G_{\mathrm{Drug}}$	0.0696 ± 0.0012	0.5379 ± 0.0035	0.6895 ± 0.0019	0.7835 ± 0.0027	0.9456 ± 0.0019	20.3739 ± 0.1757
DAPSNet $w/o\;H_{\mathrm{Pat}}{}_{i}$	0.0671 ± 0.0010	0.5327 ± 0.0022	0.6942 ± 0.0014	0.7838 ± 0.0030	0.9426 ± 0.0012	20.1247 ± 0.1799
DAPSNet $w/o\;H_{i}$	0.0657 ± 0.0012	0.5274 ± 0.0033	0.6896 ± 0.0030	0.7781 ± 0.0034	0.9375 ± 0.0006	21.4862 ± 0.1689
DAPSNet $w/o\;L_{\mathrm{DDI}}$	0.0853 ± 0.0012	0.5485 ± 0.0020	0.7003 ± 0.0023	0.7981 ± 0.0029	0.9475 ± 0.0009	22.0041 ± 0.1849
DAPSNet $w/o\;L_{IC}$	0.0679 ± 0.0013	0.5381 ± 0.0042	0.6898 ± 0.0039	0.7838 ± 0.0051	0.9398 ± 0.0009	20.3909 ± 0.2001
DAPSNet	0.0657 ± 0.0010	0.5469 ± 0.0025	0.6989 ± 0.0013	0.7942 ± 0.0024	0.9465 ± 0.0006	21.0715 ± 0.2104

#### 5.2.2 Detailed components study

In order to better illustrate the effectiveness of our model and to investigate the performance of different models for learning PR and measuring disease trajectories similarity. Specifically, we further design the ablation studies with the SOTA methods on our dataset and test on the following variants:

- GAMENet $w/o\;G_{\mathrm{DDI}}$: GAMENet model without the DDI knowledge in drug encoding.
- SafeDrug w/o Local: SafeDrug model without bipartite drug encoder.
- COGNet $w/o\;G_{\mathrm{Drug}}$: COGNet model without the EHR and DDI knowledge in drug encoding.
- DAPSNet $w/o\;G_{\mathrm{DDI}}$: DAPSNet model without the DDI knowledge in drug encoding.
- DAPSNet $w/o\;G_{\mathrm{Drug}}$: DAPSNet model without the EHR and DDI knowledge in drug encoding.
-
${\text{DAPSNet}}_{\mathrm{PatRepr}}+$ GAMENet: we replaced the PR module of GAMENet with the PR module of DAPSNet.
-
${\text{DAPSNet}}_{\mathrm{PatRepr}}+$ SafeDrug: we replaced the PR module of SafeDrug with the PR module of DAPSNet.
-
${\text{DAPSNet}}_{\mathrm{PatRepr}}+$ COGNet: we replaced the PR module of COGNet with the PR module of DAPSNet.
-
${\text{RNN}}_{\mathrm{PatRepr}}+$ DAPSNet: we replaced the PR module of DAPSNet with the PR module that adopts RNN.
-
${\text{Transformer}}_{\mathrm{PatRepr}}+$ DAPSNet: we replaced the PR module of DAPSNet with the PR module that adopts Transformer block.

Table 5 shows the results for the different variants. We conducted two sets of experiments. In the first set of experiments, we aim to explore the impact of DDI graph and EHR graph on the model performance in drug encoding. We construct different models without DDI knowledge and compare their performance. Since COGNet $w/o\;G_{\mathrm{Drug}}$ removes both the EHR graph and the DDI graph, we choose DAPSNet $w/o\;G_{\mathrm{Drug}}$ for a fair comparison. Due to the constraint of the DDI threshold (0.06), SafeDrug w/o Local reaches the lowest DDI rate. From the comparison between the results in the table and the results of the original model, it can be seen that removing the DDI graph will have a negative impact on the DDI rate and performance of the model. Meanwhile, DAPSNet $w/o\;G_{\mathrm{DDI}}$ and DAPSNet $w/o\;G_{\mathrm{Drug}}$ can maintain lower DDI rate and higher prediction accuracy compared to other variants, which shows that the whole framework is indeed effective.

**Table 5.** Performance comparison on different variant

Model	DDI	Jaccard	*F*1	PR-AUC	ROC-AUC	Avg. # of drugs
GAMENet $w/o\;G_{\mathrm{DDI}}$	0.0898 ± 0.0008	0.4824 ± 0.0025	0.6398 ± 0.0017	0.7389 ± 0.0030	0.9266 ± 0.0015	28.3004 ± 0.1141
SafeDrug w/o Local	0.0606 ± 0.0007	0.4862 ± 0.0016	0.6442 ± 0.0016	0.7423 ± 0.0013	0.9281 ± 0.0007	19.0724 ± 0.0971
COGNet $w/o\;G_{\mathrm{Drug}}$	0.0842 ± 0.0004	0.5306 ± 0.0013	0.6836 ± 0.0012	0.7706 ± 0.0013	0.9383 ± 0.0006	29.1076 ± 0.0795
DAPSNet $w/o\;G_{\mathrm{DDI}}$	0.0684 ± 0.0011	0.5435 ± 0.0028	0.6962 ± 0.0025	0.7932 ± 0.0033	0.9436 ± 0.0006	19.8746 ± 0.1534
DAPSNet $w/o\;G_{\mathrm{Drug}}$	0.0696 ± 0.0012	0.5379 ± 0.0035	0.6895 ± 0.0019	0.7835 ± 0.0027	0.9456 ± 0.0019	20.3739 ± 0.1757
${\text{DAPSNet}}_{\mathrm{PatRepr}}+$ GAMENet	0.0859 ± 0.0007	0.5104 ± 0.0033	0.6662 ± 0.0029	0.7632 ± 0.0032	0.9309 ± 0.0083	25.2817 ± 0.1604
${\text{DAPSNet}}_{\mathrm{PatRepr}}+$ SafeDrug	0.0572 ± 0.0003	0.5143 ± 0.0027	0.6748 ± 0.0026	0.7700 ± 0.0029	0.9347 ± 0.0012	21.4926 ± 0.1333
${\text{DAPSNet}}_{\mathrm{PatRepr}}+$ COGNet	0.0832 ± 0.0005	0.5382 ± 0.0024	0.6864 ± 0.0022	0.7750 ± 0.0031	0.9396 ± 0.0004	29.5331 ± 0.1248
${\text{RNN}}_{\mathrm{PatRepr}}+$ DAPSNet	0.0673 ± 0.0011	0.5205 ± 0.0033	0.6757 ± 0.0030	0.7754 ± 0.0025	0.9400 ± 0.0008	19.5021 ± 0.1698
${\text{Transformer}}_{\mathrm{PatRepr}}+$ DAPSNet	0.0682 ± 0.0005	0.5261 ± 0.0011	0.6785 ± 0.0010	0.7738 ± 0.0009	0.9405 ± 0.0006	20.0323 ± 0.1408

In the second set of experiments, we aim to investigate the differences between the PR learning modules of different models. We adopt the PR module of the DAPSNet model in GAMENet, SafeDrug and COGNet, respectively, and try the RNN-based PR module, which adopt in GAMENet and SafeDrug and the Transformer-based PR module, which adopt in COGNet into our model, respectively. We further compare the above variants with the original models. From the results, we can see that with adopting our PR module, the variants of GAMENet, SafeDrug and COGNet have significantly lower DDI rates and better accuracy compared to the model themselves. Furthermore, we replace the PR module of DAPSNet with the PR learning method of these SOTA models. There are two existing methods: one is GAMENet and SafeDrug, which use RNN to encode diagnosis and treatment information respectively, and the other is COGNet, which use Transformer block to encode the medical information separately. These two variants of DAPSNet show poor result in both DDI rate and model performance compared with DAPSNet itself. The results of the second set of experiments demonstrate that the PR learning module in our model is comprehensive and effective.

### 5.3 Case study

**Fig. 3.**
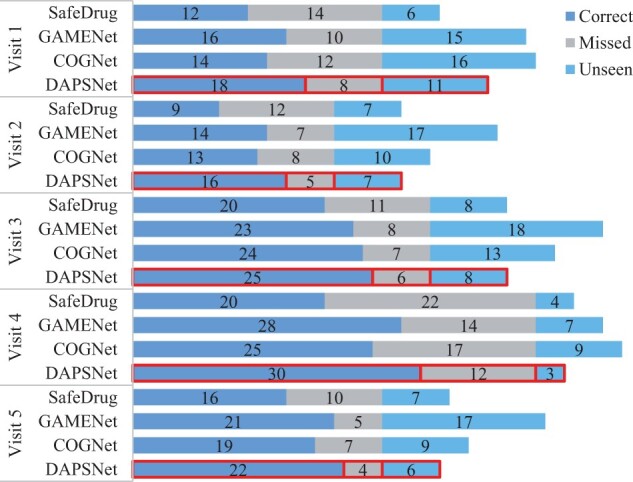
Example recommended drugs for a patient with several visits

We provide a case study to show our DAPSNet’s effectiveness. We choose a patient with five visits from the test set and use GAMENet, SafeDrug and COGNet to predict the drug combinations based on their historical medical records. The detailed diagnosis IDs, the recommended drug ATC-third code (in MIMIC-III) and drug combination DDI rate of the selected patient in each visit are provided in Table 6. In addition, we use Figure 3 for concise and intuitive display. We use ICD-9 codes to represent diagnosis records and the ATC-third codes to represent recommended drugs. Here, the ‘missed’ in Figure 3 and Table 6 indicates the drugs in the ground truth but are not predicted, while ‘unseen’ refers to the drugs predicted by the models but are not in the ground truth.

**Table 6.** Example recommended medications for a patient with several visits

Visit	Diagnosis	Method	DDI	Correct	Unseen	Missed
1	5849, 4280, 4254, V4282, 4168, 78909, 78701, 78791, 5854, 40390,49390, E9331, 5739, E8498, 42843, 7824, 4259	GAMENet	0.1083	16: N02B, A06A, B05C, A12C, C07A, C03C, N02A, A02A, B01A, J01D, A04A, A07E, R03A, D07A, N05B, J01E	15: ${A01A}^{\left(\#2\right)},\;{A02B}^{\left(\#2\right)}$, A07A, ${A12B}^{\left(\#1\right)},\;{N06A}^{\left(\#6\right)}$, A03B, ${C10A}^{\left(\#5\right)}$, C01B, ${C09A}^{\left(\#6\right)}$, N05A, ${C01D}^{\left(\#1\right)}$, R05C, ${R01A}^{\left(\#5\right)}$, C01A, C03B	10
		SafeDrug	0.0588	12: N02B, A06A, B05C, A12C, C07A, C03C, N02A, B01A, A04A, R03A, N05B, C03D	6: ${A01A}^{\left(\#1\right)},\;{A02B}^{\left(\#1\right)},\;{A12B}^{\left(\#1\right)}$, A03B, R05C, ${R01A}^{\left(\#4\right)}$	14
		COGNet	0.1023	14: N02B, A06A, B05C, A12C, C07A, C03C, N02A, A02A, B01A, J01D, A04A, R03A, N05B, C03D	16: ${A01A}^{\left(\#1\right)},\;{A02B}^{\left(\#2\right)}$, A07A, ${A12B}^{\left(\#1\right)}$, J01M, A03B, C01B, B02B, N05A, ${C01D}^{\left(\#1\right)}$, R05C, ${R01A}^{\left(\#5\right)}$, A07D, D04A, ${R05D}^{\left(\#1\right)}$, C03B	12
		DAPSNet	0.0708	18: N02B, A06A, B05C, A12C, C01C, C07A, C03C, N02A, A02A, B01A, J01D, A04A, A07E, R03A, D07A, N05B, C03D, M04A	11: ${A01A}^{\left(\#1\right)},\;{A02B}^{\left(\#1\right)}$, A12A, A07A, ${A12B}^{\left(\#1\right)}$, J01M, C01B, N05C, N05A, R05C, ${R01A}^{\left(\#4\right)}$	8
2	4280, 2859, 4019, 53081, 78701, 2874, 51881, 49390, 42841, E9331, 20400, 78650, 28800	GAMENet	0.0767	14: N02B, A02B, A06A, B05C, A12C, C07A, C03C, N02A, N05A, A04A, D07A, N05B, R01A, D04A	17: ${A01A}^{\left(\#1\right)}$, A12A, C01C, A07A, ${A12B}^{\left(\#1\right)},\;{N06A}^{\left(\#6\right)}$, A02A, J01M, ${B01A}^{\left(\#3\right)}$, C01B, H03A, N03A, ${A07E}^{\left(\#5\right)},\;{R03A}^{\left(\#2\right)}$, R05C, J01F, J01E	7
		SafeDrug	0.0617	9: N02B, A02B, A06A, B05C, A12C, C03C, N02A, J01D, R01A	7: ${A01A}^{\left(\#1\right)},\;{A12B}^{\left(\#1\right)}$, A02A, ${B01A}^{\left(\#2\right)},\;{A11C}^{\left(\#2\right)},\;{R03A}^{\left(\#2\right)},\;{M04A}^{\left(\#1\right)}$	12
		COGNet	0.1238	13: N02B, A02B, A06A, B05C, A12C, C07A, C03C, N02A, J01D, A04A, D07A, N05B, R01A	10: ${A01A}^{\left(\#1\right)}$, A07A, ${A12B}^{\left(\#1\right)}$, A02A, J01M, ${B01A}^{\left(\#3\right)}$, C01B, ${N05C}^{\left(\#2\right)},\;{A07E}^{\left(\#5\right)},\;{R03A}^{\left(\#2\right)}$	8
		DAPSNet	0.0719	16: N02B, A02B, A06A, B05C, A12C, C07A, C03C, N02A, J01D, N05A, A04A, D07A, N05B, R01A, D06B, J02A	7: A01A, A07A, A12B, J01M, ${B01A}^{\left(\#2\right)}$, C01B, ${R03A}^{\left(\#1\right)}$	5
3	5849, 40391, 4280, 78552, 99592, 42822, 2875, 99685, 2762, 0389, E8798, 5856, V5865, 4168, 79902, 51881, 49390, 0785, E9331, E8498, 42090, 4233, 4259, 27952	GAMENet	0.0808	23: N02B, A01A, A02B, A06A, B05C, A12A, A12C, C01C, A07A, C03C, A12B, N02A, A02A, N05C, J01D, A04A, R03A, D07A, N05B, D04A, D06B, J02A, J01E	18: ${N01A}^{\left(\#1\right)},\;{N06A}^{\left(\#6\right)}$, J01M, ${B01A}^{\left(\#3\right)},\;{C10A}^{\left(\#5\right)}$, C01B, D01A, ${J01X}^{\left(\#3\right)}$, B02B, ${N03A}^{\left(\#3\right)}$, N05A, D11A, ${A01A}^{\left(\#2\right)}$, A03F, R01A, V03A, L04A	8
		SafeDrug	0.0820	20: N02B, A01A, A02B, A06A, B05C, A12A, A12C, C01C, A07A, C03C, A12B, N02A, N05C, J01D, A04A, R03A, D07A, N05B, J02A, J01E	8: ${N01A}^{\left(\#1\right)},\;{N06A}^{\left(\#6\right)}$, J01M, ${B01A}^{\left(\#3\right)}$, C01B, ${A07E}^{\left(\#5\right)}$, R05C, ${R01A}^{\left(\#4\right)}$	11
		COGNet	0.0896	24: N02B, A01A, A02B, A06A, B05C, A12A, A12C, C01C, A07A, C07A, C03C, A12B, N02A, A02A, N05C, J01D, A04A, R03A, D07A, N05B, D06B, J02A, J01E, J01C	13: ${N01A}^{\left(\#1\right)},\;{B01A}^{\left(\#3\right)}$, C01B, ${C09A}^{\left(\#5\right)},\;{J01X}^{\left(\#3\right)}$, N05A, ${A07E}^{\left(\#6\right)}$, ${R01A}^{\left(\#5\right)}$, P01A, V03A, ${M04A}^{\left(\#2\right)},\;{C09C}^{\left(\#5\right)}$, J01G	7
		DAPSNet	0.0602	25: N02B, A01A, A02B, A06A, B05C, A12A, A12C, C01C, A07A, C07A, C03C, A12B, N02A, A02A, N05C, J01D, A04A, R03A, D07A, N05B, D04A, D06B, J02A, J01E, J01C	8: J01M, ${B01A}^{\left(\#2\right)}$, C01B, N05A, A07E, R05C, ${R01A}^{\left(\#5\right)}$, V03A, R05D	6
4	71946, 5849, 4280, 2859, 78039, 99685, 2763, 2761, E8798, 79092, 4168, 78959, 78909, V642, 27541, 5854, 40390, 7850, 7847, 5722, E9331, 42823, V5869, E8498, E9320, 7812, 5730, 7824, 4259, 24900, 27952	GAMENet	0.0766	28: N02B, A01A, A02B, A06A, B05C, A12C, A07A, C07A, C03C, A12B, N02A, A02A, J01M, B01A, C01B, J01D, B02B, N05A, A04A, A07E, R03A, D07A, N05B, D04A, D06B, J02A, J01E, M04A	7: ${N06A}^{\left(\#8\right)},\;{N05C}^{\left(\#4\right)},\;{J01X}^{\left(\#3\right)},\;{N03A}^{\left(\#4\right)}$, R05C, R01A, ${J05A}^{\left(\#3\right)}$	14
		SafeDrug	0.0869	20: N02B, A01A, A02B, A06A, B05C, A12C, A07A, C03C, A12B, N02A, J01M, B01A, J01D, A04A, A07E, R03A, D07A, N05B, D04A, J01E	4: ${N05C}^{\left(\#3\right)},\;{N03A}^{\left(\#3\right)}$, R05C, ${R01A}^{\left(\#4\right)}$	22
		COGNet	0.0875	25: N02B, A01A, A02B, A06A, B05C, A12C, A07A, C07A, C03C, A12B, N02A, A02A, J01M, B01A, C01B, J01D, A04A, A07E, R03A, D07A, N05B, D06B, J02A, J01E, M04A	9: ${N05C}^{\left(\#4\right)},\;{C09A}^{\left(\#7\right)},\;{J01X}^{\left(\#3\right)},\;{N03A}^{\left(\#2\right)}$, R05C, ${R01A}^{\left(\#5\right)},\;{C03D}^{\left(\#1\right)},\;{J05A}^{\left(\#3\right)},\;{J01F}^{\left(\#1\right)}$	17
		DAPSNet	0.0559	30: N02B, A01A, A02B, A06A, B05C, A12A, A12C, C01C, A07A, C07A, C03C, A12B, N02A, A02A, J01M, B01A, C01B, D01A, J01D, B02B, N05A, A04A, A07E, R03A, D07A, N05B, D04A, D06B, J02A, J01E	3: R05C, ${R03A}^{\left(\#2\right)},\;{R05D}^{\left(\#2\right)}$	12
5	4280, 4019, 4254, 42822, 99685, E8798, 79902, 49390, 4821, 34690	GAMENet	0.0910	21: N02B, A02B, A06A, B05C, A12C, A07A, C07A, A12B, J01M, B01A, N05C, J01D, D11A, A04A, A07E, R03A, D07A, R01A, D04A, D06B, J01E	17: ${A01A}^{\left(\#1\right)}$, A12A, N01A, C03C, ${N02A}^{\left(\#2\right)},\;{N06A}^{\left(\#8\right)}$, A02A, ${C10A}^{\left(\#5\right)}$, C01B, ${N03A}^{\left(\#2\right)}$, N05A, C08C, ${N05B}^{\left(\#2\right)}$, R05C, ${J05A}^{\left(\#2\right)}$, J01F, ${L04A}^{\left(\#7\right)}$	5
		SafeDrug	0.0791	16: N02B, A02B, A06A, B05C, A12C, A07A, A12B, B01A, J01D, A04A, A07E, R03A, D07A, R01A, D06B, J01E	7: ${A01A}^{\left(\#2\right)}$, C03C, ${N02A}^{\left(\#2\right)}$, C01B, N03A, ${N05B}^{\left(\#2\right)}$, R05C	10
		COGNet	0.0867	19: N02B, A02B, A06A, B05C, A12C, A07A, C07A, A12B, B01A, J01D, A04A, A07E, R03A, D07A, R01A, D06B, P01A, J02A, J01E	9: ${A01A}^{\left(\#2\right)}$, C03C, A02A, C01B, ${C09A}^{\left(\#5\right)}$, R05C, C01A, J01F, ${M04A}^{\left(\#1\right)}$	7
		DAPSNet	0.0763	22: N02B, A02B, A06A, B05C, A12C, A07A, C07A, A12B, J01M, B01A, N05C, J01D, A04A, A07E, R03A, D07A, R01A, D04A, D06B, J02A, J01E, R05D	6: ${A01A}^{\left(\#2\right)}$, C03C, ${N02A}^{\left(\#2\right)}$, C01B, ${N05B}^{\left(\#2\right)}$, R05C	4

*Note*: (${*}^{\left(\#N\right)}$) indicates that this wrongly predicted drug * has interactions with *N* correctly predicted drugs.

First, our model has the best recommendation results by comparing the results of other models with respect to each visit, our model predicts the highest number and accuracy of correct drugs, and the least number of redundant drugs. Further, we calculated the DDI score of each drug combinations. The results show that the DDI score of our model and SafeDrug predicted drugs are lower than GAMENet and COGNet due to the DDI loss function. While ensuring DDI, our model has higher accuracy than SafeDrug, While GAMENet and COGNet improve the accuracy, and the increasing number of drugs leads to higher DDI rates. We analyze the DDIs between the unseen and correct drugs predicted by each model, and interestingly, we find out that the unseen drugs predicted by DAPSNet have fewer interactions with its correctly predicted drugs than other models, providing a good constraint on the DDI rates. Combined with the previous experimental results, this case study further verifies that the drug combinations recommended by our model make a trade-off between efficacy and safety.

## 6 Conclusion

In this work, we proposed DAPSNet, a novel drug recommendation model that first integrates the historical prescription information into encoding the PR and further retrieves the patients’ different disease state similarities to jointly enhance the drug recommendation performance. Specifically, we learned the comprehensive PR from the patient’s historical visit information through a novel PR module that incorporates code- and visit-level attention mechanisms. Furthermore, we retrieve the corresponding drug combinations according to the similarity between the patients and their own and other patients’ historical disease states to obtain additional drug information, which improved the prediction accuracy. We designed an information constraint loss function based on the IB principle to constrain the PR and obtain a more robust model. The experimental results on the MIMIC-III dataset demonstrated that our DAPSNet is superior to the SOTA methods in accurately predicting drug combinations. Also, our model achieves a low DDI rate among the predicted drugs to ensure safe and effective recommendations.

## Data Availability

The data underlying this article are available in the article.
